# Concentration, purification and quantification of milk protein residues following cleaning processes using a combination of SPE and RP-HPLC

**DOI:** 10.1016/j.mex.2022.101695

**Published:** 2022-04-08

**Authors:** Christian Kürzl, Heidi Wohlschläger, Simon Schiffer, Ulrich Kulozik

**Affiliations:** Chair of Food and Bioprocess Engineering, TUM School of Life Sciences, Technical University of Munich, Germany

**Keywords:** Skim milk, Casein, Whey protein, Cleaning validation, Beta-lactoglobulin, Low protein concentration, Quality assurance, Quality control, Product safety

## Abstract

Detection and quantification of milk protein residues can be of utmost importance for validation of cleaning process efficiency in removing even traces of residues as well as quality assurance and product safety. However, currently available assays cannot provide a combination of high sensitivity and a simultaneous quantification of the individual milk proteins. Furthermore, a low protein-to-protein-variability and high compatibility with other reagents such as residual cleaning agents (e.g. surfactants) cannot be ensured. Therefore, a new method was developed comprised of a pre-concentration of proteins by solid-phase extraction and optimisation of the sensitivity of an existing reversed-phase high performance liquid chromatography method for the separate quantification of bovine milk proteins κ-Casein, α_S2_-Casein, α_S1_-Casein, β-Casein, α-Lactalbumin, and β-Lactoglobulin. Hereby, solid-phase extraction enables robust and reproducible purification and concentration of protein residues with a high protein recovery rate and flexible adjustment of concentration factors. The increased sensitivity of the reversed-phase high performance liquid chromatography method was achieved by changes in the measurement wavelength and guanidine buffer concentration. This new method enables reproducible concentration, purification and quantification of protein concentrations below 7 ng mL^−1^ and thus can be used to detect milk protein residues in highly diluted aqueous systems.

• Concentration, purification and quantification of milk protein residues with a high recovery rate of proteins (> 94%) and high reproducibility (coefficient of variation (CV) < 3.0%)
• Flexible adjustment of sample volumes allows the utilisation of high concentration factors (≤ 500) without compromising the recovery rate of proteins (recovery rate of proteins decreases by 2.74% per 100 CF)

## Graphical abstract

**Figure:**
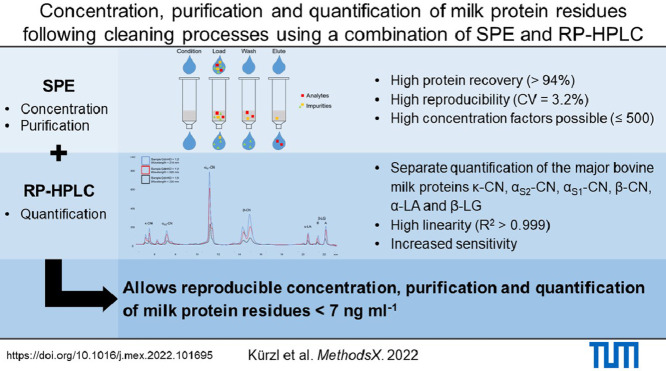

Specifications table

**Table:** 

Subject Area:	Chemistry
More specific subject area:	Protein analysis
Method name:	Concentration, purification and quantification of milk protein residues following cleaning processes using SPE and RP-HPLC
Name and reference of original method:	G. Bobe, D.C. Beitz, A.E. Freeman, G.L. Lindberg [1]: Separation and Quantification of Bovine Milk Proteins by Reversed-Phase High-Performance Liquid Chromatography, J Agric Food Chem 46 (1998) 458–463.
Resource availability:	• SPE cartridges: Phenomenex Strata C18-T cartridges, 55 µm, 140 Å (Phenomenex LTD, Aschaffenburg, Germany) • Vacuum chamber: Supelco Visiprep 12-port (Sigma-Aldrich Chemie GmbH, Taufkirchen, Germany) • RP-HPLC system ○ Agilent 1200 series chromatograph (Agilent Technologies, Waldbronn, Germany) ○ C18 analytical silica-based column: Maisch ReproSil-Pur 300 ODS-3, 4.6 × 150 mm, 5 µm (Dr. A. Maisch HPLC GmbH, Ammerbuch-Entringen, Germany) ○ Analytical guard cartridge: Agilent Zorbax 300SB-C18, 4.6 × 12.5 mm, 5 µm (Agilent Technologies, Waldbronn, Germany) ○ Agilent ChemStation software (Rev.C.01.08) • Pasteurised (74 °C, 28 s) skim milk (purchased from the local dairy Molkerei Weihenstephan, Freising, Germany) • Analytical protein standards for κ-CN, α-CN, β-CN, α-LA, β-LG B, β-LG A (Sigma-Aldrich Chemie GmbH, Taufkirchen, Germany) • Guanidine buffer (modified from Bobe et al. [1]; reagents from Merck KGaA, Darmstadt, Germany) ○ Guanidine hydrochloride (GdnHCl) ○ Trisodium citrate ○ Dithiothreitol (DTT) ○ BisTris buffer • Trifluoroacetic acid (TFA) (Merck KGaA, Darmstadt, Germany) • Acetonitrile (ACN) (Merck KGaA, Darmstadt, Germany) • Ultrapure water (from MilliQ Integral 3, Merck KGaA, Darmstadt, Germany) • Ca(OH)_2_ (Merck KGaA, Darmstadt, Germany) • NaOH (Merck KGaA, Darmstadt, Germany) • HCl (Merck KGaA, Darmstadt, Germany) • Ultrasil 08 (Ecolab Europe GmbH, Wallisellen, Switzerland) • Ultrasil 120 (Ecolab Europe GmbH, Wallisellen, Switzerland)

## Method details

### Background information and applicability of the method

The analysis of milk protein residues in highly diluted systems, as in cleaning validation or quality control, still poses a major challenge. Standard assays such as Bradford [2,3], bicinchoninic acid (BCA) [4], Coomassie-staining [5], Lowry [6], Dumas [7], or ultraviolet (UV) absorption provide easy-to-use approaches for protein concentrations in the range of 0.5–2000 µg mL^−1^ (without taking the assay-specific sample dilutions into account). Therefore, they are limited in their applicability for the validation of cleaning processes or the detection of allergens such as β-Lactoglobulin (β-LG) in production plants where significantly lower concentrations can be relevant and specific proteins need to be identified. In contrast to the firstly mentioned methods, polyacrylamide gel electrophoresis (PAGE) [8] and RP-HPLC [1,9,10] allow a simultaneous detection and quantification of individual milk proteins. For this method, RP-HPLC was chosen due to being a reproducible and well-established method for protein quantification of bovine milk samples.

However, the sensitivity of commonly used RP-HPLC methods for milk protein quantification is limited to a protein concentration of 6.25 µg mL^−1^ [1,9], which is insufficient in determining traces of milk proteins on surfaces of technical equipment in highly diluted aqueous cleaning solutions after cleaning-in-place (CIP) processes. Therefore, a solid-phase extraction (SPE) was established in this work as a concentration method prior to RP-HPLC analysis.

The working principle of the applied SPE approach is based on a hydrophobic binding of proteins onto a modified C18 silica gel embedded into a cartridge while other substances in the sample solution pass unhindered [11,12]. Besides hydrophobic binding as the main binding mechanism, remaining free silanol groups also allow for polar secondary interactions such as hydrogen bonding. Due to the working principle of SPE, it is possible to adjust the concentration factor freely by increasing the applied sample volume. Even highly diluted solutions can be concentrated to such an extent that they fall within the detection range of the quantification method with regard to the specific analyte. A limitation of the concentration by SPE is given by the loading capacity of the chosen cartridge size [11]. Compared to this approach, other concentration methods such as centrifugal filters or acetone precipitation can only achieve high concentration factors with larger centrifuges or several repetitions of the centrifugation step due to large volumes to process.

With regard to the protein binding onto the SPE material, the sample flow rate must be controlled and cannot exceed a certain limit, without a decrease in the dynamic binding capacity [11,12]. Hence, the applied sample volume determines the time required to bind the proteins in an initial phase of the concentration process on the SPE material. After hydrophobically binding the proteins on the SPE material, a washing step with ultrapure water was conducted in order to remove unbound as well as other weakly bound substances, e.g. residual cleaning agents or other sample components such as salts and lactose. Subsequently, the analytes were eluted with a low volume of a more hydrophobic solvent – containing acetonitrile (ACN), trifluoroacetic acid (TFA) and ultrapure water – from the cartridge. Hereby, the extent of hydrophobic and polar regions in the analyte as well as the amount of remaining free silanol groups determine the required hydrophobicity of the eluent. Thus, it is possible to reduce the sample volume. Due to hydrophobic regions in the structure of milk proteins, this method shows a high recovery rate of proteins over the concentration process. Due to the binding mechanisms being mainly based on hydrophobic interactions and to some extent on polar secondary interactions, the binding strength depends on the protein's structure, size and state. Hence, a change in analyte always necessitates new calibration and validation. Details will be discussed in the *Method validation* section.

In summary, the SPE-based method can be conducted to purify and concentrate analyte and to remove substances that might interfere with a quantification of the individual milk proteins by RP-HPLC. Since the applied sample volume and initial protein concentration determine the processing time of the concentration step by SPE, the available sample volume and protein concentration could restrict the applicability of this method. Therefore, an increase in the sensitivity of the subsequent RP-HPLC quantification step was investigated to reduce the necessary concentration factor, the required sample volume and the SPE time.

With regard to the protein analysis in skim milk by RP-HPLC, Bobe et al. [1] developed a method which enables the quantification of the four major caseins (CN)(κ-CN, α_S2_-CN, α_S1_-CN and β-CN) as well as the two major whey proteins (α-Lactalbumin (α-LA), and β-LG) in bovine milk. In this method, the milk proteins are diluted in a guanidine (GdnHCl) buffer and bind by hydrophobic interactions onto a silica-based C-18 RP-HPLC column. Afterwards, the proteins are gradually removed by an ACN based solvent gradient with increasing hydrophobicity, which allows a quantification of the individual bovine milk proteins by UV-detection. Hereby, the applied wavelength of the UV-detector determines the detected component in the sample. At a wavelength of 260–290 nm mostly the aromatic amino acids Tryptophan, Tyrosine, Histidine and Phenylalanine are detected, whereas peptide bond absorption is detected at wavelengths of 190–230 nm, respectively [13], [14], [15]. Thus, at low wavelength the absorption is dominated by peptide bonds. At these wavelengths, aromatic side chains show only a slight absorption which induces a lower protein-protein variability due to the reduced impact of the content of specific amino acids. However, technical difficulties were reported for detection at wavelengths below 200 nm, such as an increasing signal noise induced by the absorption of air [13]. Furthermore, absorption maxima shift for different proteins and their different molecular states, depending on pre-treatment, degree of nativity as well as present solvents and their concentrations [13], [14], [15], [16]. Therefore, depending on the sample matrix and the aim of the analysis, different wavelengths might be required, which is an unknown, thus limiting the reliability of the results for samples with different protein states.

Due to the impact of changes in solvents and sample composition, different observations regarding the performance optima at different wavelengths were made by several authors [1,9,10,17]. Bobe et al. [1] used a wavelength of 220 nm instead of 210 nm within the UV-detection during RP-HPLC, in order to reduce baseline noise as well as to enhance the peak resolution. In contrast to that, Bonfatti et al. [9] applied a wavelength of 214 nm instead of 220 nm for the same reasons. However, in a subsequent study, Dumpler et al. [10] showed a high resolution with a detection wavelength of 220 nm, similar to the observations made by Bobe et al. [1]. Besides wavelength, the RP-HPLC method for milk protein quantification as established by Bobe et al. [1] has been further adapted by Bonfatti et al. [9], Bonizzi et al. [17] and Dumpler et al. [10] regarding the optimisation criteria of higher resolution, increased simplicity of sample preparation and quantification of protein concentrates. The lowest detectable protein amount for an individual protein was reported to be approximately 0.5 µg for α-LA (6.25 µg mL^−1^) [1,9]. In the latest modification of the method, as reported by Dumpler et al. [10], a minimum quantifiable individual protein concentration of approximately 25 µg mL^−1^ in the initial sample can be determined. This is due to the maximum injection volume of most HPLC setups being limited to 100 µl, as well as the 5-fold dilution with guanidine buffer as part of the sample preparation prior to the analysis. The most recent modification of the method, conducted by Dumpler et al. [10] enables a reduction of the sample analysis time, as well as a high resolution by an optimisation of the applied solvent gradient. Additionally, the buffering capacity was enhanced by an increased sodium citrate amount and furthermore, the storage stability at room temperature was increased by an enhanced guanidine concentration (5.1 M) during sample preparation. The guanidine concentration was increased in order to counterbalance the instability of whey proteins at room temperature as observed by Bonfatti et al. [9]. The instability of whey proteins was assumed to be caused by a guanidine concentration insufficient to completely denature the present whey proteins and thus leading to a refolding of the proteins in the buffer solution as a function of the storage time, explaining the decreased detection of proteins during RP-HPLC after storage of samples for several hours at room temperature [10,18]. With this increased GdnHCl concentration (5.1 M) in the sample, proteins in concentrated skim milk with up to 27% total solids could be quantified.

However, the dilution of milk samples with GdnHCl buffer as shown by Dumpler et al. [10] was calculated for concentrated samples, whereas the approach investigated in this study focuses on the quantification of protein residues in diluted aqueous systems and thus protein concentrations lower than a third of that in milk after pre-concentration with SPE. The concentration by SPE, as explained above, produces a purified aqueous protein system with low protein content. Therefore, lower buffering capacity and lower GdnHCl concentrations are required in comparison to the concentrations used by Dumpler et al. [10]. Hence, the sample dilution in GdnHCl buffer could be reduced to 1:2 leading to a final GdnHCl concentration of 3 M in the sample. Furthermore, due to the reduced dilution of the initial sample, the detection limit is decreased. Additionally, a wavelength of 214 nm was applied to increase signal response and thus decrease the detection limit.

The combined improvement of SPE and RP-HPLC methods, as targeted in this study, allows to concentrate, to purify and to quantify proteins in diluted test samples containing extremely low amounts of milk protein residues. The developed method enables a flexible adjustment of required concentration factors according to the target analyte under consideration. In this regard, the increase of applied sample volume and thus concentration factor is only limited by the loading/binding capacity of the SPE cartridge, which must not be exceeded. Furthermore, the specific binding mechanism of proteins onto the SPE material reduces the appearance of interfering substances, e.g. of polar substances such as residual cleaning agents, in the produced concentrate. The developed SPE method is likely to be applicable to other protein systems apart from diluted aqueous solutions derived from removing milk protein deposits on solid surfaces or similar applications, possibly with slight adjustments to the procedure or sample state. A main limitation of the method is that higher concentration factors (CF) and volumes are accompanied by longer processing times of up to 11 h. In future studies, a significant reduction of processing times might be achieved by using larger cartridges that allow higher flow rates or pre-concentrating the sample with e.g. a rotary evaporator.

### Preparation of solvents

Solvent A (0.1% TFA, 90% ultrapure water and 10% ACN) and solvent B (0.07%TFA, 90% ACN and 10% ultrapure water) are prepared according to Dumpler et al. [10] and used for the RP-HPLC analysis. Furthermore, the solvents A and B are also used during the sample preparation and elution step of the SPE concentration. The guanidine buffer is prepared according to Dumpler et al. [10] containing 6 M guanidine hydrochloride, 21.5 mM trisodium citrate, 19.5 mM dithiothreitol (DTT) and 0.1 M BisTris buffer (pH 6.8).

### Concentration and purification of sample solution by SPE

The assessment criteria for the applicability of the developed SPE method is determined by the recovery rate of proteins (see Eq. (1)). It is defined as the quotient of the detected protein concentration after SPE ($c_{\text{protein},\;\text{detected}}$) and the applied protein concentration of the initial sample solution without SPE ($c_{\text{protein},\;\text{applied}}$), measured by RP-HPLC. Hereby, it can be differentiated between the total protein concentration and the concentration of an individual milk protein.

(1) $$\text{Recovery}\;\text{rate}\;\text{of}\;\text{protein}\;=\;\frac{c_{\text{protein},\;\text{detected}}}{c_{\text{protein},\;\text{applied}}}$$

The CF produced by SPE is defined as the quotient of the applied initial sample volume $V_{\text{sample}}$ and the elution volume $V_{\text{elution}}$ during protein desorption (see Eq. (2)).

(2) $$\text{CF}\;=\;\frac{V_{\text{sample}}}{V_{\text{elution}}}$$

The applied concentration factor achievable by SPE depends on the availability of sample volume as well as the protein concentration in the sample. If the protein concentration in the sample is known or can reliably at least roughly be estimated, the required CF and sample volume can be calculated with the following Eqs. (3) and (4):

(3) $$CF_{\text{req}}\;=\;\frac{c_{\text{min},\;\text{RP}-\text{HPLC}}}{c_{\text{protein},\;\text{applied}}}\cdot \frac{1}{\text{protein}\;\text{recovery}\;\text{rate}}$$

with $c_{\text{min},\;\text{RP}-\text{HPLC}}$ as the lowest quantifiable protein concentration of the RP-HPLC method (see *Method validation* section) and $CF_{\text{req}}$ as the required concentration factor to process a quantification with RP-HPLC. Combining Eqs. (2) and (3), the required sample volume ($V_{\text{sample},\;\text{req}.}$) critical for a concentration with SPE in order to conduct a protein quantification with the modified RP-HPLC method, can be calculated by the following Eq. (4):

(4) $$V_{\text{sample},\;\text{req}.}\;=\;\frac{c_{\text{min},\;\text{RP}-\text{HPLC}}}{c_{\text{protein},\;\text{applied}}}\cdot \frac{V_{\text{elution}}}{\text{protein}\;\text{recovery}}=CF_{\text{req}.}\cdot V_{\text{elution}}$$

Here, $V_{\text{elution}\;}=\;3\;\text{mL}$ and values for $c_{\text{min},\;\text{RP}-\text{HPLC}}$ can be seen in Table 2.

If the sample volume is limited or contains various proteins, the required sample volume for the concentration factor must be calculated for the protein with the lowest concentration or a certain target protein. If the sample volume is not limited, it is recommended to aim for a $V_{\text{sample}}\;>\;1.1\;\cdot \;V_{\text{sample},\;\text{req}.}$.

In case that the analyte concentration is unknown, the execution of several SPEs with different sample volumes is recommended to determine whether an overloading of the SPE material occurred. An overloading of the material is indicated by a decrease in recovery as a function of an increasing sample volume. The loading capacity depends on sample composition and chosen material type and amount. However, to estimate the value of the binding capacity, approximately 1–5% of the sorbent amount can be used according to manufacturers. With the SPE cartridges used in this study containing 500 mg sorbent, the binding capacity equals approximately 5–25 mg protein. An overloading of the SPE material could not be determined in this study for protein amounts < 6 mg. Besides the supposedly maximal cartridge loading capacity of below 25 mg protein, the application of 1–6 mg protein onto the cartridge is recommended since it maintains a safety margin for other unwanted substances also blocking parts of the cartridge's loading capacity.

### Procedure of the SPE method

#### Sample preparation

(1) If applicable: filter particulates from sample with a sterile filter (0.45 µm)
(2) Add 22.67% of a 3 mM Ca(OH)_2_ solution and 8% solvent B to the sample
(3) Adjust pH with 1 M HCl and 1 M NaOH to 7.0 ± 0.03

*Note*: The increase in hydrophobicity, achieved by addition of solvent B, was necessary to improve protein binding onto the sorbent. Low binding with pure water as a medium could be due to proteins assembling into more hydrophilic complexes or insufficient wetting of the silica surface. Ca(OH)_2_ was added to reduce negative charges in the calcium-sensitive proteins α_S2_-CN, α_S1_-CN and β-CN [19,20] and thus improve their binding onto the hydrophobic sorbent.

#### SPE cartridge preparation

(1) Connect the cartridge to the vacuum chamber and open the connection between cartridge and vacuum chamber (outlet)
(2) Condition the sorbent with 6 mL Acetonitrile (slight vacuum can be applied; flow rate < 3.0 mL min^−1^)
(3) Equilibrate the sorbent with 6 mL ultrapure water (slight vacuum can be applied; flow rate < 3.0 mL min^−1^)

*Note*: The flow rate should be <3.0 mL min^−1^ for all steps except the washing step (< 6.0 mL min^−1^) and can be adjusted by partially closing the outlet or applying a slight vacuum. In case the next step (sample application) is not executed immediately afterwards, leave ∼1 mL ultrapure water in the cartridge and close the outlet until continuation of the process to avoid a drying of the SPE sorbent.

#### Sample application

(1) Apply the desired amount of prepared sample volume (at least the minimum required volume calculated with Eq. (4)) before the ultrapure water from the equilibration step has completely run through, to avoid a drying of the cartridge (slight vacuum can be applied; flow rate < 3.0 mL min^−1^)
(2) Rinse the cartridge with 6 mL ultrapure water

*Note*: Avoid drying of the SPE material and the entrapment of air bubbles. In case the next step (washing application) is not executed immediately afterwards, leave ∼1 mL ultrapure water in the cartridge and close the outlet until continuation of the process to avoid a drying of the SPE sorbent.

#### Washing step

(1) Wash with 100 mL ultrapure water (slight vacuum can be applied), with a flow rate of < 6.0 mL min^−1^
(2) Apply vacuum and let the cartridge run dry for 5 min to remove any leftover water
(3) Close the outlet

#### Elution step

(1) Apply 1.5 mL of elution agent containing 61.6% solvent A and 38.4% solvent B and let it soak for 3 min to ensure diffusion of the elution agent into the sorbent matrix and to enhance the removal of analytes from the sorbent
(2) Open the outlet (no vacuum applied; flow rate < 3.0 mL min^−1^)
(3) After the passage, apply a vacuum for 15 s to recover the elution agent and thus enable a reproducible quantification of the protein amount of the initial sample
(4) Repeat steps 1–3 once

*Note*: The optimum solvent composition and thus hydrophobicity depends on sample characteristics, cartridge characteristics and interactions between the two. The chosen composition showed the highest recovery for diluted milk samples compared to more polar or more hydrophobic eluents. It is known that a repeated elution with low volumes leads to an enhanced protein recovery rate compared to a single elution with a higher volume. For diluted skim milk samples, a 2-fold elution with 1.5 mL each showed a protein recovery rate of 94.2%, while keeping the elution volume low and therefore enabling concentration factors of up to 500 (see *Method validation* section for details). Due to the incomplete recovery, SPE cartridges should not be re-used. Complete recovery of the elution agent can be validated with a 100 µl pipette.

#### Calculation

Calculate the protein mass of the sample applied to the SPE cartridge $m_{\text{protein},\;\text{applied},\;\text{SPE}}$ by RP-HPLC measurement (see Eq. (5)) and the inclusion of the additional SPE terms (see Eqs. (1) and (2)).

(5) $$m_{\text{protein},\;\text{applied},\;\text{SPE}}\;=\;c_{\text{protein},\;\text{initial}\;\text{sample}}\cdot V_{\text{sample}}=\frac{c_{\text{protein},\;\text{applied},\;\text{RP}-\text{HPLC}}\;\cdot \;V_{\text{elution}}}{\text{protein}\;\text{recovery}\;\text{rate}}$$

with $c_{\text{protein},\;\text{applied},\;\text{RP}-\text{HPLC}}$ as the protein concentration detected in RP-HPLC and $c_{\text{protein},\;\text{initial}\;\text{sample}}$ as the protein concentration in the initial sample.

To calculate whether a selected sample volume is within the compatible range of RP-HPLC and SPE, the protein recovery rate should be omitted since proteins that bind only weakly and are washed out or bind too strong to be eluted, must still be counted towards the loading capacity.

#### Validation

(1) Process an RP-HPLC of a skim milk sample in order to obtain the protein concentration for the following validation
(2) Dilute skim milk 1:120, 1:900, 1:3000, 1:6000 and 1:9000 with ultrapure water, conduct a concentration by SPE (as described above), dilute the SPE eluate 1:2 in GdnHCl buffer and process a RP-HPLC with a detection wavelength of 214 nm
(3) Evaluate results according to Eq. (6) and calculate the protein recovery rate according to Eq. (1).

*Note*: If this method is to be applied to other protein systems, the concentration step by SPE might require an adjustment. If the protein recovery rate is low, analyse the sample volume that passed the SPE column in the loading step with RP-HPLC to obtain if protein can be found in this passed sample (in this case, the binding strength is too low). If not, binding strength might be too high or elution strength too low. In this case, the elution agent, elution mode and pre-treatment might require further adjustments.

### Quantification with RP-HPLC

#### General procedure

(1) Prepare a 1:2 mixture of the eluted sample from the SPE in the GdnHCl buffer
(2) Let the solution react at room temperature (RT) for 30 min
(3) Conduct an RP-HPLC analysis as described by Dumpler et al. [10]. Set the detection wavelength to 214 nm and the injection volume to 100 µl

*Note:* After incubation with GdnHCl buffer for 30 min, conduct RP-HPLC within 24 h to exclude decreasing protein detection due to refolding of proteins (see Method validation section).

#### Calibration of the RP-HPLC method

(1) Dilute protein standards κ-CN, α-CN and β-CN 1:9, α-LA 1:5, β-LG B and β-LG A 1:3 separately in GdnHCl buffer.
(2) Process an RP-HPLC measurement with an applied wavelength of 214 nm in the UV detector for each individual standard, with an injection volume of 5 µl, 10 µl and 20 µl. Subsequently, correlate the obtained peak areas with applied protein amounts (taking purity of purchased standards into account).

#### Quantification

(1) Separate and integrate RP-HPLC-peaks of the different milk proteins κ-CN, α_S2_-CN, α_S1_-CN, β-CN, α-LA and β-LG as shown by Dumpler et al. [10]
(2) Calculate the applied protein concentration according to Eq. (6):
  (6) $$c_{\text{protein},\;\text{applied},\;\text{RP}-\text{HPLC}}\;=\;c_{\text{protein},\;\text{detected},\;\text{RP}-\text{HPLC}}\;\cdot DF_{\text{RP}-\text{HPLC}}\cdot \frac{V_{\text{inj},\text{cal}}}{V_{\text{inj},\;\text{actual}}}$$

with $c_{\text{protein},\;\text{detected},\;\text{RP}-\text{HPLC}}$ as the protein concentration detected in RP-HPLC, $DF_{\text{RP}-\text{HPLC}}$ as the dilution factor (DF) of the sample with GdnHCl buffer (DF = 2), $V_{\text{inj},\text{cal}}$ as the calibrated injection volume (20 µL) and $V_{\text{inj},\;\text{actual}}$ as the applied injection volume (100 µL).

#### Validation

(1) Dilute skim milk 1:7.5, 1:10, 1:15, 1:20, 1:30, 1:50, 1:75, 1:100, 1:250, 1:400, 1:600, 1:750, 1:1000 in ultrapure water. Dilute the obtained solutions 1:2 in GdnHCl buffer.
(2) Conduct an RP-HPLC measurement (with a detection wavelength of 214 nm) with an injection volume of 50 µl and 100 µl. and compare detected RP-HPLC amounts with applied protein amounts.

## Method validation

### SPE procedure

#### Reproducibility and protein recovery rates of the SPE method

**Fig. 1:**
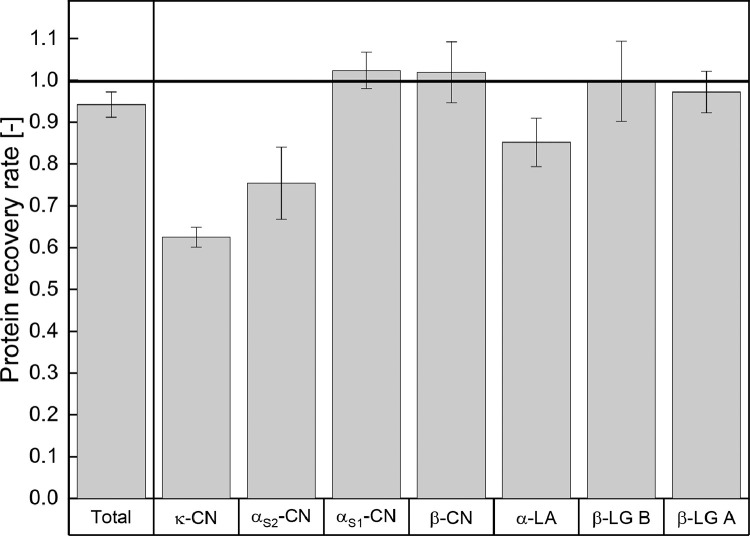
Protein recovery rates after SPE and RP-HPLC for the total protein as well as for the individual milk proteins (κ-CN, αS2-CN, αS1-CN, β-CN, α-LA, β-LG B and β-LG A) (*n* = 8).

Protein concentration with the described protocol for SPE results in a total protein recovery of 94.2 ± 3.0% (*n* = 8). A high reproducibility of the applied SPE method could be proven by a calculation of the standard deviation (3.0%) and the CV (3.2%) of the total protein recovery rate between experiments. Furthermore, the protein recovery rates of the individual milk proteins were calculated and are shown in Fig. 1. Compared to the recovery rate of the total protein concentration, α_S1_-CN, β-CN, β-LG B and β-LG A achieved higher recovery rates with 102.4 ± 4.3%, 101.9 ± 7.3%, 99.8 ± 9.5% and 97.2 ± 5.0%, respectively. In contrast to that, the recoveries of κ-CN, α_S2_-CN and α-LA were lower, with 62.5 ± 2.3%, 75.4 ± 8.6% and 85.2 ± 5.8%, respectively.

### Influence of residual cleaning agents on protein recovery rate

**Fig. 2:**
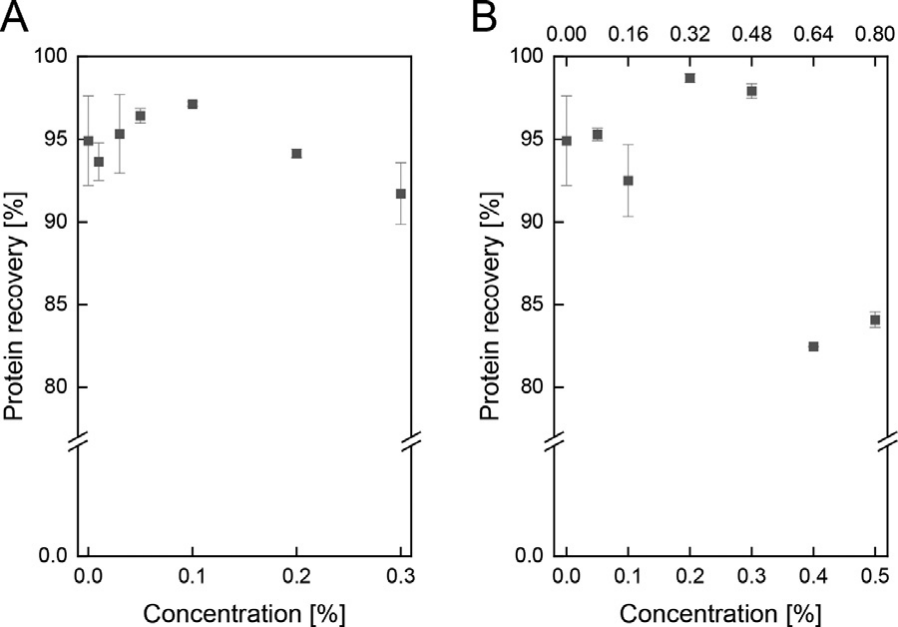
Protein recovery of total milk proteins for varying residual concentrations of cleaning agents NaOH (A) and a combination of industrial cleaning agents Ultrasil 120 (upper x-axis) as an alkaline NaOH-based cleaning agent and Ultrasil 08 (lower x-axis) as a surfactant-based additive (B).

Furthermore, it was investigated whether residual cleaning agents in the sample can affect the affinity of proteins to the SPE sorbent. It should be noted, that for cleaning validation and thus the detection of protein residues after cleaning and rinsing steps, concentrations of residual cleaning agents are nonetheless expected to be low. To examine the influence of cleaning agents on protein recovery and thus the robustness of the method, different concentrations were added to diluted milk samples (1:200 in water) for 20 min at 50 °C to resemble common cleaning conditions. Afterwards, pH was neutralized, and SPE followed by RP-HPLC was processed. The resulting influence of chemical concentrations on protein recovery is depicted in Fig. 2. This study validated the robustness of the method for NaOH (A) and a common combination of an industrial alkaline NaOH-based cleaner (Ultrasil 120) with a surfactant-based additive (Ultrasil 08) (B). No clear trend can be observed for protein recoveries with NaOH concentrations up to 0.3%, and no significant differences from NaOH-free samples were detected. Hence, NaOH-induced changes in protein charge or denaturation do not deteriorate the protein recovery for NaOH concentrations up to 0.3%. On the contrary, for industrial cleaning agents with an alkaline cleaner and a surfactant-based additive, three different levels of protein recovery can be observed. No difference to chemical-free samples can be noted for low concentrations up to 0.1%/0.16% (of Ultrasil 08 and Ultrasil 120). For medium concentrations of 0.2%/0.32%–0.3%/0.48%, protein recovery increases to 98.7% and 97.9%, respectively. At higher concentrations of 0.4%/0.64%–0.5%/0.8%, protein recovery decreases to 82.5% and 84.1%, respectively. Consequently, it can be concluded that the SPE method is more susceptible to influences on the binding affinity when using industrial cleaners that, next to NaOH, contain additives such as surfactants. Accordingly, separate validation needs to be conducted for different cleaning agents and concentrations.

### Influence of sample volume and protein concentration

In order to validate the applicability of the described method to different sample volumes and protein concentrations, the effect of the protein concentration in the sample as well as sample volume or process time on protein recovery rate was investigated. Therefore, skim milk was diluted with ultrapure water and processed by SPE (applied protein concentrations of 294.97 µg mL^−1^, 38.03 µg mL^−1^, 11.41 µg mL^−1^, 5.71 µg mL^−1^ and 3.80 µg mL^−1^ in the SPE sample). To compensate the different protein concentrations, the applied sample volumes have been adjusted accordingly to recover identical protein amounts (5.7 mg in total) after SPE and RP-HPLC. With constant flow rates of 3 mL min^−1^, the changes in sample volume induce longer sample process times on the one hand and higher CFs on the other hand. An overview of the influence of skim milk dilution on sample volume, protein concentration and thus CF and sample processing time can be seen in Table 1.

**Table 1:** Influence of different dilution factors of skim milk (1:120, 1:900, 1:3000, 1:6000 and 1:9000; details see Method details section) on resulting sample volume, SPE volume, protein concentration, CF and sample processing time.

Samplenumber	Sample volume[mL]	SPE volume^a^[mL]	Protein concentration^b^[µg mL^−1^]	CF^c^ [-]	Sample processing time^d^[min]
1	20	26.1	294.97	6.7	8.7
2	150	196.1	38.03	50.0	65.4
3	500	653.6	11.41	166.7	217.8
4	1000	1307.3	5.71	333.3	435.7
5	1500	1960.9	3.80	500.0	653.5

a sample volume after addition of SPE ingredients.

b total protein concentration in the sample after addition of SPE ingredients.

c calculated based on the initial sample volume without SPE additives and the elution volume of 3.0 mL.

d calculated with a flow rate of 3.0 mL min^−1^; only refers to the process time of the applied sample, no preceding or succeeding SPE steps included.

**Fig. 3:**
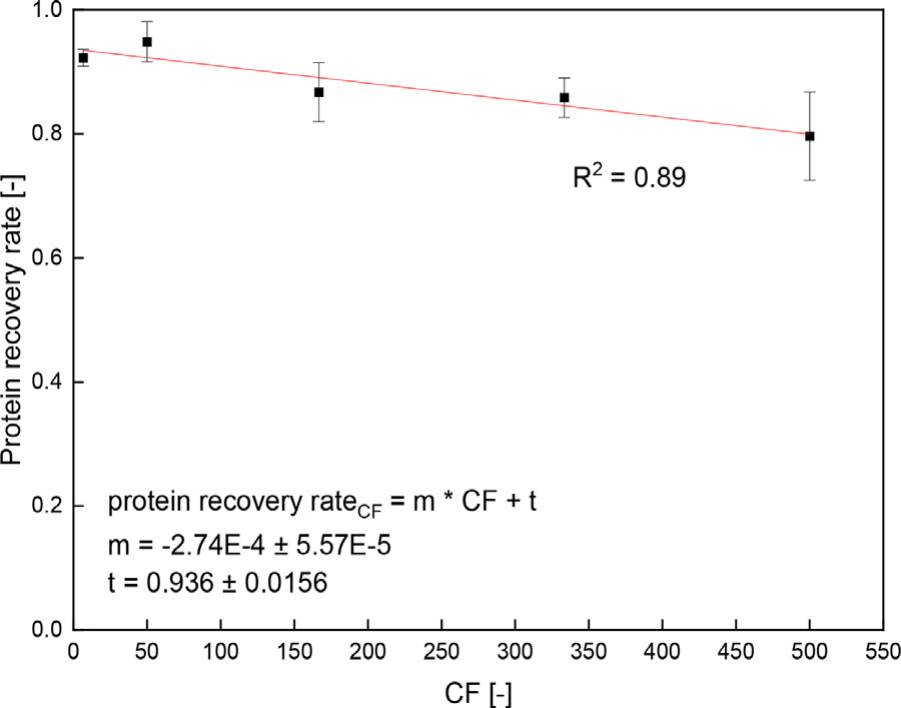
Protein recovery rate of the total protein for skim milk samples diluted 1:120, 1:900, 1:3000, 1:6000 and 1:9000 after concentration by SPE and quantification by RP-HPLC.

In addition to the process time of samples with different protein concentrations and thus volumes, the protein recovery rates of the different samples in dependence of the CF were calculated (see Fig. 3 and Eq. (7)). The protein recovery for a CF = 50 corresponds to the results shown in Fig. 1. In Fig. 3 it can be seen that a significant linear decrease (*p* < 0.05) occurs with increasing CF and thus sample volume. Hereby, the correlation coefficient R^2^ = 0.89 indicates a linear correlation between the protein recovery rate and the CF of the sample, and one-way ANOVA confirms the significance of the slope (*p* < 0.05).

Furthermore, the linear Eq. (7) shows that the negative slope and thus decrease in protein recovery rate with increasing sample volume equals 2.74% per 100 CF or 9.12% per 1000 mL sample volume.

(7) $$\text{protein}\;\text{recovery}\;\text{rat}e_{\text{CF}}\;=\;-2.74E-4\;\cdot \;\text{CF}\;+\;0.936$$

Therefore, the protein recovery rate remains high for large values of CF. This allows flexible scalability of sample volume and CF. While CF is not limited to 500, using Eq. (3) with an exemplary CF of 500, the corresponding protein recovery rate of 79.6% and the minimum quantifiable protein concentration with RP-HPLC for e.g. the allergen β-LG B of 3.30 µg mL^−1^ shows that protein concentrations c_β-LG B_ < 0.0083 µg mL^−1^ can be quantified with this approach.

### Validation of the modified RP-HPLC method

In order to examine the effects of wavelength changes on peak separation and base line noise, diluted skim milk was diluted 1:2 in GdnHCl buffer and analysed by RP-HPLC with a UV-detector wavelength of 214 and 220 nm. Furthermore, the effect of changes in the GdnHCl concentration in skim milk samples was investigated by a comparison of diluted skim milk diluted 1:2 and 1:5 in GdnHCl and measured by RP-HPLC (detector wavelength = 214 nm).

**Fig. 4:**
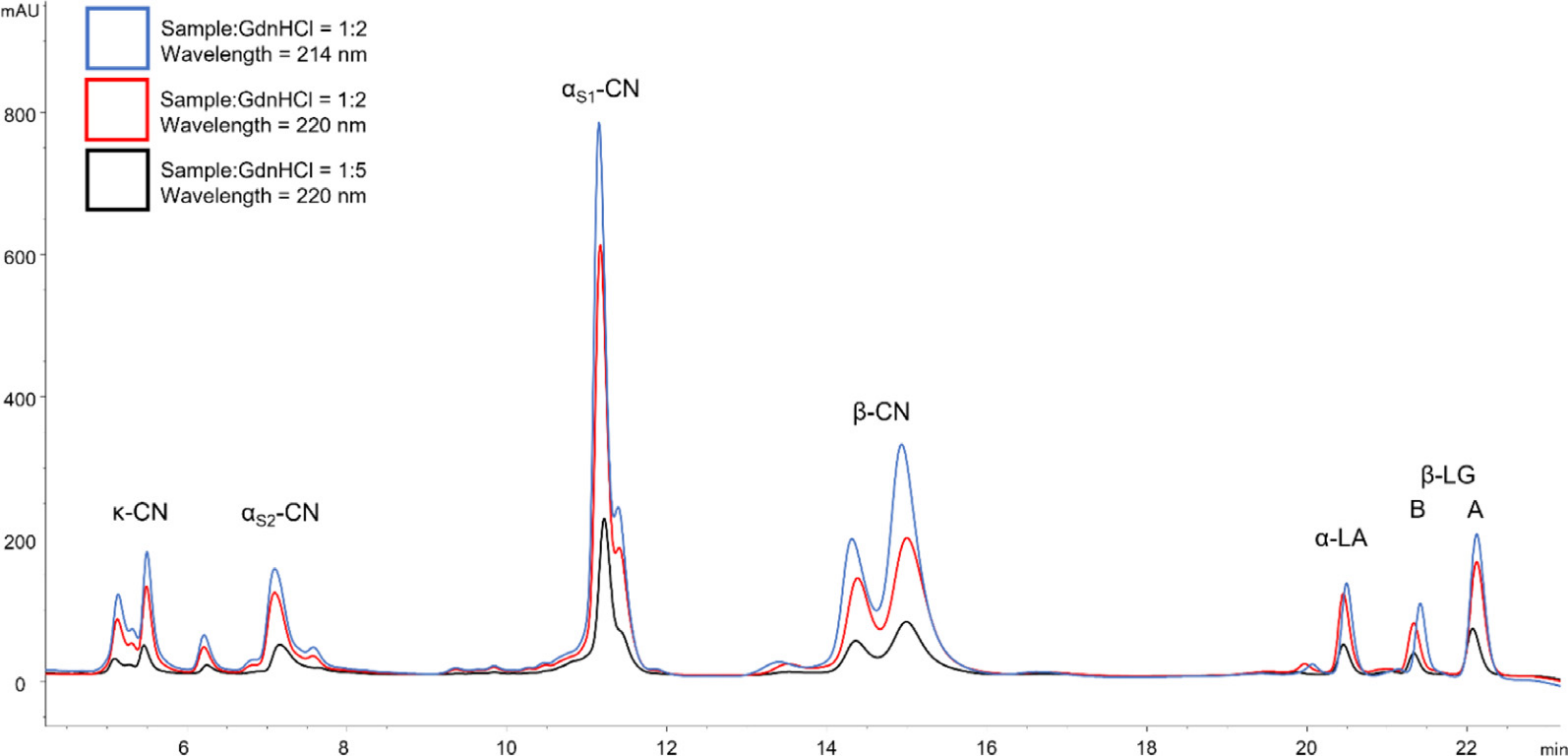
UV signal of a RP-HPLC analysis with a 1:5 sample dilution in GdnHCl-buffer and a detection wavelength of 220 nm as postulated by Dumpler et al. [10] (black); a 1:2 sample dilution in GdnHCl-buffer and a detection wavelength of 220 nm (red); a 1:2 sample dilution in GdnHCl buffer and a detection wavelength of 214 nm (blue).

In Fig. 4 it can be seen that a reduction in the GdnHCl buffer concentration from 1:5 to 1:2 increases the detected peak areas by 126%. Additionally, it could be shown that a change of the detection wavelength from 220 nm, as applied by Dumpler et al. [10], to 214 nm does not affect baseline noise or peak resolution but increases the signal response and thus the peak area by 37% for diluted skim milk samples. In summary, the applied changes in wavelength and GdnHCl buffer concentration can improve the method postulated by Dumpler et al. [10] regarding the detected peak area by 209%. Therefore, the applied changes facilitate peak identification and integration of low protein content samples and thus enable a lowering of the detection limit which reduces sample volumes required in SPE. Total protein amounts in skim milk were found to be 34.70 ± 1.18 mg mL^−1^ (*n* = 14) which is in accordance with literature values [1,21,22]. Thus, it can be concluded that the changes applied to the RP-HPLC method are not affected by additional noise or protein loss due to incomplete denaturation followed by refolding, as it was observed by Bonfatti et al. [9], and yields correct protein values.

**Fig. 5:**
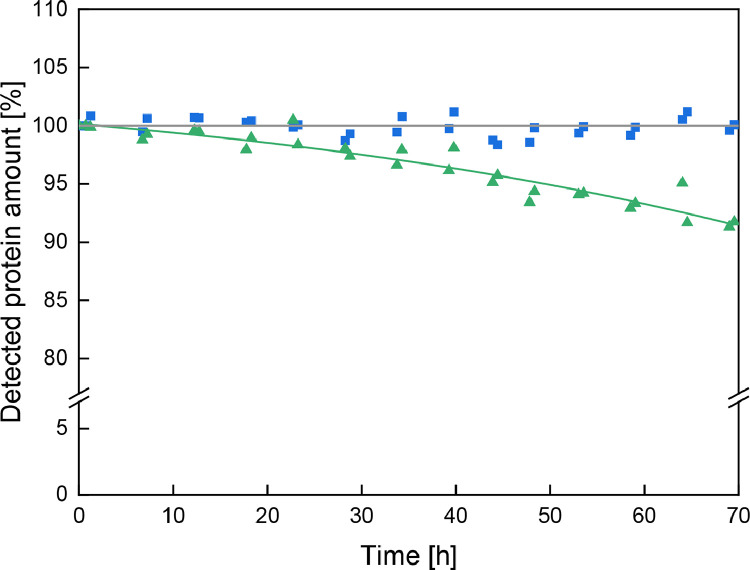
Detected amount (with RP-HPLC) of whey proteins (green triangles) and caseins (blue squares) as a function of the storage time at RT relative to the initial protein amount detected after 30 min incubation with GdnHCl buffer. The grey line highlights the reference point of 100% detected protein.

Furthermore, a refolding and thus decreased detection during RP-HPLC due to incomplete denaturation had to be excluded. For this purpose, the storage stability of samples was analysed. After 30 min incubation of diluted milk samples (1:200 in water) with GdnHCl buffer, samples were stored at RT and repeatedly analysed with RP-HPLC for 70 h. The detected protein amounts, relative to the initial value after incubation and summed for whey proteins and caseins, are depicted in Fig. 5. For caseins, no trend deviating from the initial value can be observed within the examined time frame of 70 h. For whey proteins, a slightly decreasing protein amount can be observed over time, mainly attributed to β-LG A. Hence, it can be concluded that the reduced GdnHCl-buffer concentration in the sample does lead to incomplete denaturation and thus refolding over time. Nevertheless, the refolding-related decrease in detected protein amounts is less than 2% after 24 h and less than 5% after 48 h allowing sufficient analysis time.

**Fig. 6:**
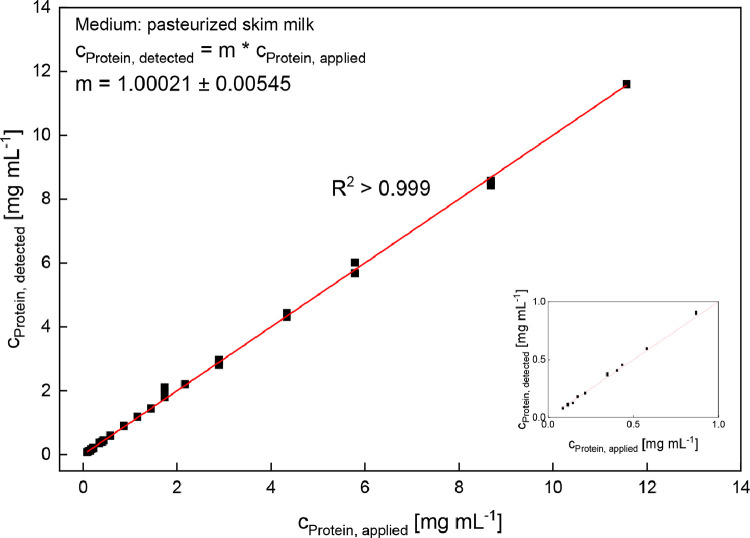
Detected protein concentration (with RP-HPLC) as a function of the applied protein concentration (0.09 mg mL^−1^–11.57 mg mL^−1^). The applied concentration was calculated according to Eq. (6).

To examine the linear working range of the method, pasteurised skim milk was diluted to different extents (1:7.5–1:1000; for details see *Method details* section), mixed with GdnHCl buffer and analysed by RP-HPLC. The detected total protein concentration $c_{\text{Protein},\;\text{detected}}$ in skim milk as a function of the applied protein concentration $c_{\text{Protein},\;\text{applied}}$ was used to investigate the linearity of the slope with the correlation coefficient as well as the significance of the slope by ANOVA (see Fig. 6).

Within the examined range of protein concentrations of up to 11.57 mg mL^−1^ total protein, a slope significantly different from zero (*p* < 0.05) could be confirmed by ANOVA. Furthermore, the correlation coefficient was determined to be R^2^ > 0.999 confirming linearity within this range. The linear Eq. (8) shows that the slope *m* = 1.00021 ± 0.00545 is close to one, implying that any increase in $c_{\text{Protein},\;\text{applied}}$ causes an equivalent increase in $c_{\text{Protein},\;\text{detected}}$.

(8) $$c_{\text{Protein},\;\text{detected}}\;=\;1.00021\;\cdot \;c_{\text{Protein},\;\text{applied}}$$

Besides the linear range for the total protein concentration in skim milk (see Fig. 6), the individual milk proteins (κ-CN, α_S2_-CN, α_S1_-CN, β-CN, α-LA, β-LG B and β-LG A) were also analysed for linearity within the examined range of diluted skim milk concentrations (see Table 2). Here, α-LA showed the lowest R^2^ value of 0.991 while κ-CN and α_S1_-CN showed the highest R^2^ values of 0.998. In addition to R^2^ values exceeding 0.99 for each milk protein, ANOVA confirmed a slope significantly different from zero (*p* < 0.05) for each milk protein. Linearity can therefore be assumed in the given range for individual milk proteins as well as the total protein.

**Table 2:** Test for linearity of RP-HPLC method with pasteurised skim milk.

Milk protein	Range of concentrations[µg mL^−1^]	Range of amounts^a^[µg]	R^2^^b^
κ-CN	9.6–1283.9	0.96–128.39	0.998
α_S2_-CN	8.8–1179.8	0.88–117.98	0.995
α_S1_-CN	27.5–3666.6	2.75–366.66	0.998
β-CN	27.0 –3597.2	2.70–359.72	0.997
α-LA	2.9–393.3	0.29–39.33	0.991
β-LG B	3.3–439.5	0.33–43.95	0.996
β-LG A	7.6–1017.9	0.76–101.79	0.997
**Total**	**86.8–11,566.7**	**8.68–1156.67**	**> 0.999**

a applies for an injection volume of 100 µl.

b R^2^, correlation coefficient: high values indicate linear correlation. probability for zero slope of line all < 0.001.

Overall, the performed approach enables robust and reproducible concentration, purification and concentration of bovine milk proteins in aqueous diluted skim milk samples and in presence of cleaning agents. The developed SPE method allows high protein recovery rates with flexible scalability of the CF while improvements towards the RP-HPLC method provide additional sensitivity, reducing the necessary CFs and thus processing time. However, changes in sample composition, analyte of interest or protein state will necessitate new calibration, validation and possibly adjustments to the method procedure.
